# Towards a Mathematical Formalism for Semi-stochastic Cell-Level Computational Modeling of Tumor Initiation

**DOI:** 10.1007/s10439-015-1271-1

**Published:** 2015-02-11

**Authors:** F. J. Vermolen, R. P. van der Meijden, M. van Es, A. Gefen, D. Weihs

**Affiliations:** 1 Delft Institute of Applied Mathematics, Delft University of Technology, Delft, The Netherlands; 2 Department of Biomedical Engineering, Tel Aviv University, 69978 Tel Aviv, Israel; 3 Faculty of Biomedical Engineering, Technion–Israel Institute of Technology, 3200003 Haifa, Israel

**Keywords:** Tumor initiation, Cell-based modeling, Immune system response, Fundamental solutions, Cell proliferation, Mutation, Cell migration

## Abstract

A phenomenological model is formulated to model the early stages of tumor formation. The model is based on a cell-based formalism, where each cell is represented as a circle or sphere in two-and three dimensional simulations, respectively. The model takes into account constituent cells, such as epithelial cells, tumor cells, and T-cells that chase the tumor cells and engulf them. Fundamental biological processes such as random walk, haptotaxis/chemotaxis, contact mechanics, cell proliferation and death, as well as secretion of chemokines are taken into account. The developed formalism is based on the representation of partial differential equations in terms of fundamental solutions, as well as on stochastic processes and stochastic differential equations. We also take into account the likelihood of seeding of tumors. The model shows the initiation of tumors and allows to study a quantification of the impact of various subprocesses and possibly even of various treatments.

## Introduction

Cancer, manifested as a malignant tumor, is a family of diseases that involve abnormally increased cell growth and proliferation, where cells may also detach and invade other of the body. In modern society more than 10 million new cases of cancer occur on a yearly basis, and these cancers, which vary in nature and in appearance in the body, are a major cause of death of humans. The most often occurring cancers are lung cancer, breast cancer, prostate cancer to mention just a few. In aging populations, mutations leading to cancer are more likely to take place and therefore cancer treatment, aiming at curing patients, as well as prolonging life expectancy or improvement of life quality, will set a heavier (economic) burden on health care in the future. To improve treatments and make them more efficient, scientific research is necessary to characterize disease etiology, expected behaviors, and their causes.

Tumor initiation and growth can lead to loss of normal organ function and at well-developed stages often pose serious threats to life, its quality and expectancy. Although treatment of tumors exist, not all treatments are successful in removing all threats, and thereby they aim at prolonging and increasing the quality of life. It is well-known that tumors involve abnormal cell division and proliferation often with the added ability to invade and spread into other parts of the body. The first tumor cells in a body typically result from the mutation in the genetic material (DNA) of the cell that may occur during division and/or growth. As part of the mutation, the progenitor cell and all daughter cells become immortal. Those immortal cells exhibit a new phenotype in terms of cell stiffness, proliferation rates, and significant reduction in cell death. As mutations accumulate, a tumor may remain dormant and initiate and only grow at later stages. The process, time-evolution and location of cancer initiation, growth and spreading is known to be influenced by age, gender, patient weight and patient life-style.

In order to improve current treatment of cancer, it is essential to understand the mechanisms of tumor initiation and growth. This understanding requires well-conducted experiments, which can be understood by linking them to hypotheses. This link between hypotheses and experiments clearly needs a quantification step, which is obtained through mathematical modeling, and therefore mathematical modeling has become an important step in the understanding of tumor dynamics. Regarding mathematical models for tumor growth, many different models exists. Most of these models are based on solving partial differential equations for densities of tumor cells, where large size tumors can be dealt with, see among many others, for instance studies by Refs. 1,2,13,18. Some of these models are based on sudden localized changes of the tumor cell density where in fact a moving boundary problem is solved, often using a phase-field approach, such as the Cahn–Hilliard equation, see Ref. 6. Next to these large-scale and fully deterministic approaches, there exists various small-scale (semi-)stochastic methods such as cellular automata-like models, see Refs. 9,12 or cell-based models.

The present paper is based on the use of a cell-based approach, where individual cells are considered. This formulation has similarities to the particle models that are common in many physical sciences where for instance flow problems are simulated by the treatment of individual molecules or most recently by the usage of *super molecules*, which take clusters of molecules into account. Our approach was initiated by Refs. 4,10,14,20,21, and later adopted in a very interesting papers by Refs. 3,17. The present model utilizes a domain of computation which is initially filled with epithelial cells, that may mutate during division into tumor cells, resulting in the subsequent spread and proliferation of tumor cells at the expense of the host organ cells. We also take into account the immune response mechanism by considering T-cells that transmigrate through adjacent small blood vessels and strive to kill the tumor cells. The paper deals with the development of the model which, we believe, can be used more generally to relate tumor initiation stages and progression under drug treatment, or with patient life-style and nature of the tumor cells.

The paper is organized as follows. First we present the mathematical model which is based on analytic solution types. Subsequently, we present the numerical solution technique. Then, we consider some experimental outcomes and the experimental procedures regarding tumor cells penetrating stiff tissues. Finally, we present simulation outcomes where some of the parameters are varied.

## Materials and Methods

In this section, we describe the mathematical model and the numerical method.

### The Mathematical Model

We describe the mathematical model in terms of the various processes involved. The processes are basically given by diffusion of cytokines, migration of cells (random walk, chemotaxis, haptotaxis and passive migration), proliferation and death of cells, immune cell engulfment of tumor cells, mutation of epithelial cells, and extravasation of T-cells out of small blood vessels. The treatment of the processes is described in separate sections.

Since we consider various cell types in the present paper, we divide the set of all cells, being $n^{c}$ in number, and given by ${\mathbb{V}}(t) = \{1,\ldots ,n^{c}\}$, note that as a result of proliferation and death, the total number of cells evolves in time, including the tumor cells, T-cells and epithelial cells. This set is divided into the non-overlapping subsets ${\mathbb{W}}(t)$, ${\mathbb{T}}(t)$, which respectively denote the set of T-cells (white blood cells) and tumor cells. The remainder of the cells are the constituent cells (epithelial cells) denoted by ${\mathbb{E}}(t)$. Hence ${\mathbb{V}}(t) = {\mathbb{E}}(t) \cup {\mathbb{T}}(t) \cup {\mathbb{W}}(t)$, and ${\mathbb{E}}(t) \cap {\mathbb{T}}(t) = \emptyset$,* etc.*

#### Diffusion of Cytokines

The tumor cells secrete chemokines, cytokines that induce chemotaxi, that are detected by the receptors on the surface of the T-cells. To model attraction of immune cells, the concentration gradient of the tumor-cell released chemokines is needed at the position of the T-cells. We assume that diffusion drives the spreading of the chemokines and that the production is active at the center of the tumor cell which is allowed to migrate through the domain of computation. Let ${\mathbf{x}}_j(t)$ be the position of the $j$th cell, which is a tumor cell, and let $\gamma _j(t)$ be the amount of chemokine produced per unit of time in mol/mm^3^/h, then the concentration of chemokines in mol/mm^3^ satisfies

1 $$\begin{aligned}&\displaystyle {\frac{\partial c}{\partial t} - D \Delta c = \gamma _j(t) \delta ({\mathbf{x}} - {\mathbf{x}}_j(t)),} \qquad j \in {\mathbb{T}}(t) \\&c(t,{\mathbf{x}}) = 0. \end{aligned}$$

In the above equation, $\Delta (.) = \nabla \cdot \nabla (.)$ represents the Laplace operator. This above equation is based on treating the release of chemokines by tumor cells as a point source. This treatment allows to extend the cytokine distribution to include multiple tumor cells. The set of tumor cells is represented by ${\mathbb{T}}(t)$. Then the above equation upon taking into account the active tumor cells is extended to

2 $$\begin{aligned}&\displaystyle {\frac{\partial c}{\partial t} - D \Delta c = \sum _{j\in {\mathbb{T}}(t)} \gamma _j(t) \delta ({\mathbf{x}} - {\mathbf{x}}^T_j(t)),} \\&c(t,{\mathbf{x}}) = 0. \end{aligned}$$

Equation (1) is solved formally by application of the fundamental solution (Green’s Function) and the Duhamel principle and the solution is represented by

3 $$\begin{aligned} c(t,{\mathbf{x}}) = \int _0^t \frac{\gamma (s)}{[4 \pi D (t-s)]^{d/2}} \exp \left(-\frac{|| {\mathbf{x}} - {\mathbf{x}}^T_j(s) ||^2}{4 D (t-s)}\right) ds, \end{aligned}$$

see Evans^7^ for more details. Here $d$ represents the dimensionality. Although this representation requires the integration over an increasing time-interval, it is, however, generic regarding dimensionality. In addition, it allows the evaluation of the concentration at any point we want, whereas when the use of discretisation techniques such as the finite-element method necessitates the computation of the concentration over a mesh of points over the entire domain of computation. A drawback is the fact that the above solution holds over the entire space and that the diffusion coefficient $D$ has to be constant over space. A time-varying diffusion coefficient can be dealt with easily. Linearity of the diffusion equation allows the application of the Superposition Principle to extend solution Eq. (3) to multiple sources from tumor cells

4 $$\begin{aligned} c(t,{\mathbf{x}}) = \sum _{j\in {\mathbb{T}}(t)} \int _0^t \frac{\gamma (s)}{[4 \pi D (t-s)]^{d/2}} \times \exp\left (-\frac{|| {\mathbf{x}} - {\mathbf{x}}_j(s) ||^2}{4 D (t-s)}\right) ds. \end{aligned}$$

We evaluate the concentration gradient at the positions of the T-cells, which chase the tumor cells by the mechanism of chemotaxis; this will be described in a later section. The determination of the gradient of the concentration is straightforward by differentiation with respect to the spatial coordinates. There is the following minor complication we still have to deal with: Imagine that tumor cell $k$ has died, but then the chemokines released by it are still dispersed throughout the tissue. Hence the contribution to the total chemokine concentration field cannot be ignored. For this reason we have to store each tumor cell that ‘ever lived’ in the set of tumor cells that release chemokines. This implies that Eq. (4) has to be extended with the entries of tumor cells that lived but died afterwards. For these tumor cells, the time-interval during which they lived is recorded by $[\tau ^{\rm B}_k,\tau ^{\rm D}_k]$ where $\tau _k^{\rm B}$ and $\tau _k^{\rm D}$, respectively, denote the time of birth and death of tumor cell $k$. This set of dead tumor cells is denoted by ${\mathbb{T}}_{\rm D}(t)$, hence Eq. (4) is adjusted to

5 $$\begin{aligned} \displaystyle {c(t,{\mathbf{x}}) = \sum _{j\in {\mathbb{T}}(t)} \int _0^t \frac{\gamma (s)}{[4 \pi D (t-s)]^{d/2}} \exp \left(-\frac{|| {\mathbf{x}} - {\mathbf{x}}_j(s) ||^2}{4 D (t-s)} \right) ds+} \\ \displaystyle {\sum _{k\in {\mathbb{T}}_{\rm D}(t)} \int _{\tau ^{\rm B}_k}^{\tau _k^{\rm D}} \frac{\gamma (s)}{[4 \pi D (t-s)]^{d/2}} \exp \left(-\frac{|| {\mathbf{x}} - {\mathbf{x}}^k(s) ||^2}{4 D (t-s)} \right) ds.} \end{aligned}$$

We note that the concentration that has been determined using the Green’s Fundamental Solution represents a chemical signal where the T-cells will migrate towards its positive gradient. Specifically, this migration can result from chemotaxis, haptotaxis, or their combination, where, respectively cells move up the gradient of chemical attractant existing in the surrounding fluid, or up the gradient of the number of adhesion sites in the extracellular matrix, see Ref. 11 for instance.

#### Migration of Cells

Several mechanisms induce the migration of cells: chemotaxis, contact forces, haptotaxis and random walk. The migration component induced by the cell-cell and cell-wall contact forces is referred to as *passive migration* in this paper. Another component could be mechanical drag, which is neglected in the present study. The haptotaxis kinetics are modeled using the formalism outlined in Ref. 20. For completeness, we repeat the most important steps. Consider a set of generic cells with spatial positions ${\mathbf{x}}_j(t)$ at a certain time $t$. Then, it is hypothezied that the migration of the cells is determined by the experienced gradient of the strain energy density. To this extent, we simplify the Green’s Functions by considering an exponential attenuation of the strain energy density from the pulling forces exerted by cell $j$ with radius $R_j$ on position ${\mathbf{x}}_j$:

6 $$\begin{aligned} M({\mathbf{x}}) = M_j^0 \exp \left( -\lambda _j \frac{||{\mathbf{x}} - {\mathbf{x}}_j ||}{R_j}\right), \end{aligned}$$

where the induced strain energy density $M_j^0$ is given by

7 $$\begin{aligned} M_j^0 = \frac{ F_j^2 }{ 2 \pi ^2 E_{\rm s} R_j^4 }. \end{aligned}$$

Here $F_j$ and $E_{\rm s}$, respectively, denote the force exerted by cell $j$ and the elastic modulus of the extracellular matrix. This strain energy density is detected by the other cells provided the value exceeds a certain threshold. Since the scalar quantity energy is additive, to get the total strain energy density as a result of all the cells, say $n^{\rm c}$, that mechanically pull the extracellular matrix, we get

8 $$\begin{aligned} M({\mathbf{x}}) = \sum _{j=1}^{n^{\rm c}} M_j({\mathbf{x}}_j(t)) = \sum _{j=1}^{n^{\rm c}} M_j^0 \exp \left( -\lambda _j \frac{||{\mathbf{x}} - {\mathbf{x}}_j ||}{R_j} \right). \end{aligned}$$

The cells detect the above given strain energy density function. A second important contribution to the strain energy density is that induced by physical contact between neighboring cells. We note that this approach necessitates the simplification that the effects of residual stresses on the mechanical behavior of the extracellular matrix are neglected.

**Figure 1:**
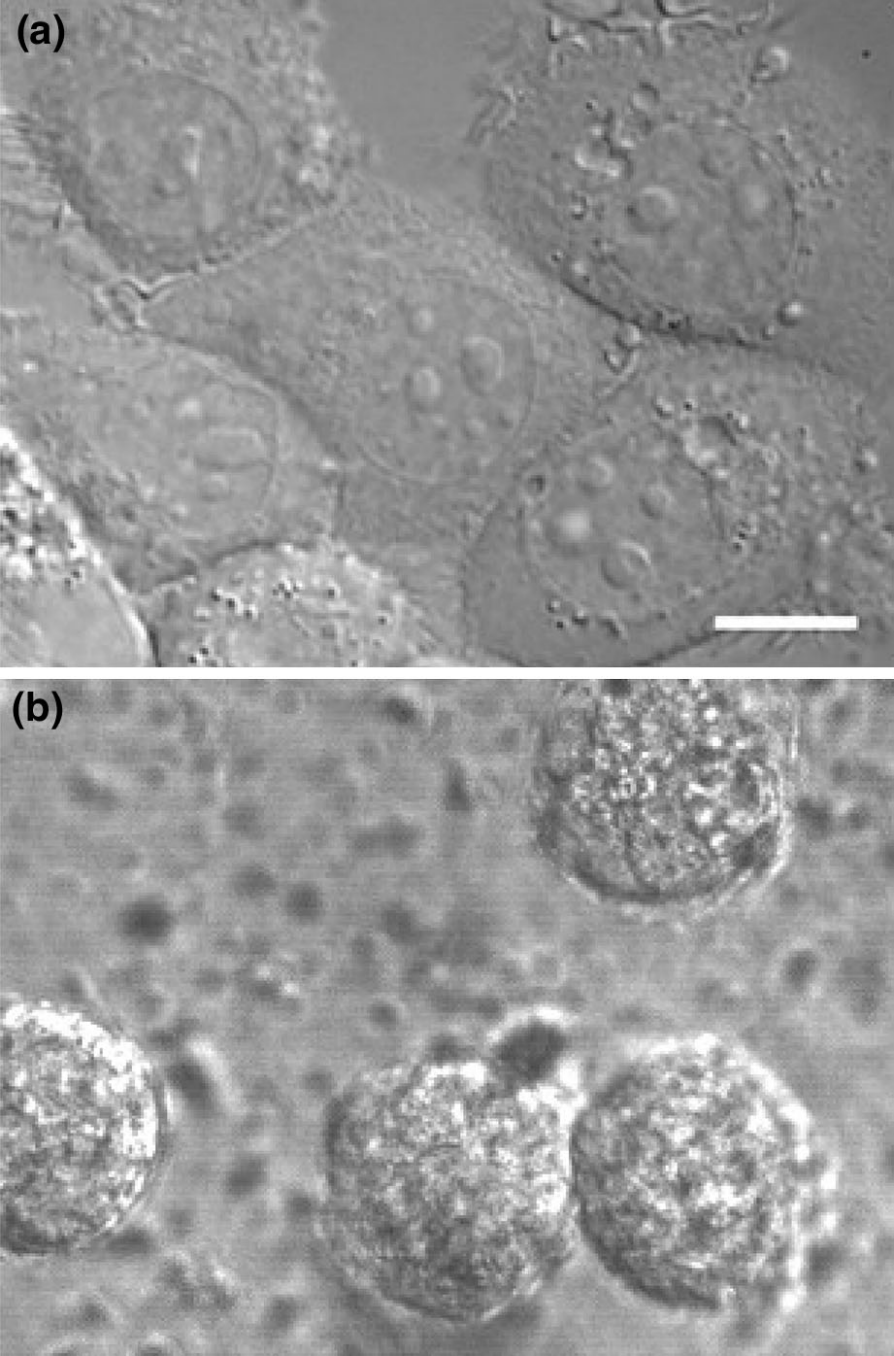
Cells that pack closely may affect morphology. a Metastatic breast cancer cells (MDA-MB-468) on glass substrate, dense packing results in polygonal shapes. b On a soft, physiological, cross linked collagen substrate, metastatic breast cancer cells (MDA-MB-231) remain rounded. Scale bar is 10* μ*m.

Densely packed cells will induce a deformation in the each cell’s morphology, in epithelial cells typically resulting in polygonal shapes (see Fig. 1a), where tumor cells with specific mutations may also lose that shape. However, in a physiological environment cells are observed to be rounded many times (Fig. 1b). Hence, we assume that all cells to remain spherical or to maintain a circular projection on a 2D substrate.

Let the characteristic penetration depth of cell $i$ into cell $j$ be given by

9 $$h = \frac{1}{2} \max (0,R_i+R_j- || {\mathbf{x}}_j - {\mathbf{x}}_i ||),$$

where $R_i$ and $R_j$ are the cell radii. Using Hertz’ model for contact forces, see Refs. 8,20, and integration over the strain to get the strain energy density, we obtain for the contribution of cell $i$ pushing on cell $j$:

10 $$\begin{aligned} M^{ij} = {\left\{ \begin{array}{ll} \displaystyle {\frac{16}{25} \frac{E_{c}}{\sqrt{2} \pi } \left( \frac{h}{R_i} \right) ^{5/2}}, &{} { \text { in } {\mathbb{R}}^2} \\ \displaystyle {\frac{2}{5} \frac{E_{\rm c}}{\sqrt{2} \pi } \left( \frac{h}{R_i} \right) ^{5/2}}, &{} { \text { in } {\mathbb{R}}^3}\\ \end{array}\right. } \end{aligned}$$

where $E_{\rm c}$ denotes the elastic modulus of the cells. The above relation gives the intercellular-contact contribution to the strain energy density that is responsible for repelling cells from one-another if cells partly overlap. Similar rules are used to model the cell-outer boundary of the domain and for the contact forces between the cell and the wall of a small blood vessel. All the contact contributions will make the cell move away from the body it is in contact with. The strain energy that is experienced by the cells from other distant cells will make the cell move towards regions of higher strain energy density. This is the reason why contributions from long-distance haptotaxis will be assigned the positive sign and all other contributions from contact mechanics will be given a negative sign.

To this extent, migration of cell $i$ is directed towards increasing values of the strain energy density and its magnitude is determined by the actual value of the strain energy density that the cell experiences. The magnitude is adjusted in order to only account for those contributions that exceed a certain threshold that was experimentally observed in Refs. 5,16. The adjustment gives

11 $$\begin{aligned} M({\mathbf{x}}) = \sum _{j=1}^{n^{\rm c}} M_j({\mathbf{x}}_j(t)) = \sum _{j=1}^{n^{\rm c}} M_j^0 \exp \left( -\lambda _j \frac{||{\mathbf{x}} - {\mathbf{x}}_j ||}{R_j} \right) {\mathbb{1}}_{|| {\mathbf{x}} - {\mathbf{x}}_j(t) || < L }({\mathbf{x}}), \end{aligned}$$

where ${\mathbb{1}}$ denotes the indicator function, which, here, is defined by

12 $$\begin{aligned} {\mathbb{1}}_{\varOmega }({\mathbf{x}}) = {\left\{ \begin{array}{ll} 1, &{} {\mathbf{x}} \in \varOmega , \\ 0, &{} {\mathbf{x}} \notin \varOmega . \end{array}\right. } \end{aligned}$$

The direction of the migration velocity direction is given by

13 $$\begin{aligned} {\mathbf{z}}_i = \displaystyle {\sum _{j=1_{j \ne i}}^{n^{\rm c}} M_j({\mathbf{x}}_i(t)) {\mathbf{v}}_{ij}(t)}, \text {for all } i \in \{1,\ldots ,n\}, \end{aligned}$$

where the ${\mathbf{v}}_{ij}$ denotes the unit vectors that connect a pair of cells, which are given by

14 $$\begin{aligned} {\mathbf{v}}_{ij} = \frac{ {\mathbf{x}}_j - {\mathbf{x}}_i }{ || {\mathbf{x}}_j - {\mathbf{x}}_i || }. \end{aligned}$$

Finally, we normalize the migration vector by

15 $$\begin{aligned} {\hat{{\mathbf{z}}}}_i = \frac{{\mathbf{z}}_i}{|| {\mathbf{z}}_i ||}. \end{aligned}$$

Since the displacement over a time frame is assumed to be in the direction of ${\mathbf{z}}_i$ where the magnitude of the displacement is assumed to be proportional to the strength of the mechanical signal, we have

16 $$\begin{aligned} {\mathbf{x}}_i(t+\Delta t) - {\mathbf{x}}_i(t) = \Delta t \alpha _i M({\mathbf{x}}_i) {\hat{\mathbf{z}}}_i, \qquad \text {for all } i \in \{1,\ldots ,n\}, \end{aligned}$$

where $\alpha _i$ is a parameter with a dimension $\left[ \frac{m^2 s}{kg} \right] = \left[ \frac{m^3}{N s} \right]$, in which the force is directed along $\varOmega$, hence perpendicular to the upward cell traction force $F$. This cell substrate friction force directed along $\varOmega$ is denoted by $f$. Since dead cells are not able to migrate actively according to chemical or remote mechanical signals, the physical condition of a cell, that is, its viability, is an additional parameter that influences the ability of a cell to migrate. The parameter $\alpha _i$ should also contain the cell viability since the cell mobility depends on the cell viability. Therefore, we express $\alpha _i$ by

17 $$\begin{aligned} \alpha _i = \left( \frac{F_i}{\hat{F}}\right) ^2 \beta _i \frac{R^3}{f}. \end{aligned}$$

Herewith, we write the deterministic mechanical part (composed of haptotaxis and contact forces) by

18 $$\begin{aligned} d {\mathbf{x}}_i(t) = \alpha _i M({\mathbf{x}}_i(t)) {\hat{\mathbf{z}}}_i dt, \qquad i \in {\mathbb{V}}(t). \end{aligned}$$

The above described considerations were originally presented in Ref. 20. We remark that an alternative formulation could be obtained by a direct evaluation of the strain energy density where one could use the direct (numerical) solution or its corresponding Green’s Functions. Since the last-mentioned approach induces slower calculations, we adopt the present formalism in this paper.

Next to mechanically driven cell migration, we consider the contribution resulting from chemotaxis, which is assumed to be significant for the T-cells that chase the tumor cells. The concentration of tumor-secreted chemokines was computed in the previous subsection and this concentration is subsequently substituted into the equation of motion of the T-cells:

19 $$\begin{aligned} d {\mathbf{x}}_i = \alpha _i M({\mathbf{x}}_i(t)) {\hat{\mathbf{z}}}_i dt + \mu \nabla c(t,{\mathbf{x}}_i) dt, \qquad i \in {\mathbb{W}}(t), \end{aligned}$$

where $\mu$ denotes the chemotactic sensitivity parameter. Next to chemotaxis, and mechanotaxis, cells are known for exhibiting random walk, this is incorporated by a vector-Wiener process, $d {\mathbf{W}}(t)$, which is distributed normally with zero mean and variance $dt$. The formal definition can be found in, for instance.^19^ This gives the following stochastic differential equation for cell migration

20 $$\begin{aligned} d {\mathbf{x}}_i(t) = {\left\{ \begin{array}{ll} \alpha _i M({\mathbf{x}}_i(t)) {\hat{\mathbf{z}}}_i dt + \sigma d {\mathbf{W}}(t), \text { for } i \in {\mathbb{V}}(t) \setminus {\mathbb{W}}(t), \\ \alpha _i M({\mathbf{x}}_i(t)) {\hat{\mathbf{z}}}_i dt + \mu \nabla c(t,{\mathbf{x}}(t)) + \sigma d {\mathbf{W}}(t), \text { for } i \in {\mathbb{W}}(t). \end{array}\right. } \end{aligned}$$

We note that^10^ adopt similar stochastic differential equations for modeling cellular migration. The above equation can be generalized easily to incorporate other biological signals that influence the migration of cells. We remark that the present paper aims at an introduction of a generic model incorporating biomechanical and biochemical signals. From a qualitative point of view, the introduction of additional signals does not complicate the problem from a mathematical point of view.

#### Proliferation, Death, Cell Cycle and Tumor Spreading

Proliferating cells are known to roughly go through the following stages: G1. cell growth by increase of cytoplasm, S. copying of DNA, G2. cellular growth and M. cell division (mitosis). To this extent, we incorporate the increase of volume of the cell during the proliferation process. We assume that the cells are actively migrating in the G1, S and G2-phases. During the final proliferative phase, that is the M-phase, we assume that the cells do not migrate actively, they will only migrate as a result of contact forces exerted by neighboring cells. Let $R_j$ denote the radius of cell $j$, then we assume the following relation

21 $$\begin{aligned} d R_j = \gamma _j \kappa dt + \sigma _g dW(t), \end{aligned}$$

where $\kappa$ denotes a growth constant and $\sigma _g$ takes into account probabilistic variations due to uncertainties in tissue composition, cell composition, access of necessary chemicals,* etc.* Since the cell only grows actively in the G1 and G2 phase, the $\gamma$-parameter is determined by the phase a cell is in:

22 $$\begin{aligned} \gamma _j = {\left\{ \begin{array}{ll} 1, \qquad \text { if cell j is in the G1 or G2-phase}, \\ 0, \qquad \text { else.} \end{array}\right. } \end{aligned}$$

The growth parameter mimics the average growth behavior and since the expectance $E(dW(t)) = 0$, we use

23 $$\kappa = \frac{R_{\rm max} - R_0}{T_{\rm G1}+T_{\rm G2}},$$

where $R_0$ and $R_{\rm max}$, respectively, denote the initial (minimal) and the average radius under mitosis. The expected time-interval length during the S-phase is denoted by $E(T^{\rm S}_i) = T^{\rm S}$. Let us assume that the time-step is constant and given by $\Delta t$, and let $N$ be the number of time-steps then the simulated time is given by $t_N = N \Delta t$. Let $P_{S\rightarrow {\rm G2}}(\Delta t)$ denote the probability that a cell transforms from phase S into G2 over a time-interval $\Delta t$. Then, the likelihood that cell $i$ has a residence time of $t_N$ in the S-phase is given through the geometric distribution

24 $${\mathbb{P}}(T^{\rm S}_i = N \Delta t) = ( 1 - P_{S\rightarrow {\rm G2}}(\Delta t) )^{N-1} P_{S\rightarrow {\rm G2}}(\Delta t).$$

The expectancy of the number of time-steps in the S-phase is given by

25 $$E(N) = \frac{1}{P_{S\rightarrow {\rm G2}}(\Delta t) }.$$

It follows that the expected residence time of cell $i$ in the S-phase is given by

26 $$\begin{aligned}&{E(T^{\rm S}_i) = \lim _{N\rightarrow \infty } \sum _{n=1}^N t_n {\mathbb{P}}(T^{\rm S}_i = n \Delta t) =} \\&{ \lim _{N\rightarrow \infty } \sum _{n=1}^N n \Delta t ( 1 - P_{S\rightarrow {\rm G2}}(\Delta t) )^{n-1} P_{S\rightarrow {\rm G2}}(\Delta t) = \Delta t E(N) = \frac{\Delta t}{ P_{S\rightarrow {\rm G2}}(\Delta t) }}. \end{aligned}$$

Upon setting $E(T_i^{\rm S}) = T^{\rm S}$, the above result is rewritten as

27 $$P_{S\rightarrow {\rm G2}}(\Delta t) = \frac{\Delta t}{T^{\rm S}}.$$

This is an important result since now we can relate the probability of transition from the S-phase to the G2-phase over a certain time-step $\Delta t$ to the average residence time in the S-phase and the time-step used. We assume that mitosis takes place immediately after the G2-phase. The position of the daughter cell is determined as presented in Vermolen & Gefen with an extension to three spatial dimensions, where a random orientation-direction between the mother-and daughter is selected. Mother-and daughter cell are subsequently displaced along this direction such that the point of physical contact coincides with the center of the mother cell.

Regarding cell death, similar probabilistic principles are applied. We compute the likelihood that a cell dies during a time-step $\Delta t$. Initially, we assume that the death of the cell is not affected by its environment. On the basis of the average time-interval of the cell cycle, with length $T^{\rm C}$, we estimate the probability of death during time-interval $\Delta t$. We relate this probability as well as possible to possible experimental observations. In experimental colonies, one could follow the dynamics of doubling of the population. Knowing the cell-cycle for proliferation, we can relate the idealized cell proliferation kinetics and the actual experimentally observed population dynamics to the kinetics of cell death (or apoptosis in some cases). Let $P_{\rm D}(\Delta t)$ denote the probability that a cell dies within the interval $\Delta t$, the probability that a cell dies within a time-interval $t_N = N \Delta t$ should not depend on the magnitude of the time-step employed. Hence we hypothesize that the probability of survival of a cell over the time-interval $[0,t_n]$ is given by $1-P_{\rm D}(n\Delta t) = (1 - P_{\rm D}(\Delta t))^n$. Imagine that the length of this time-interval is given by the average time-span of the cell cycle, given by $T^{\rm C}$, that is $t_n = T^{\rm C}$, then

28 $$1 - P_{\rm D}(T^{\rm C}) = (1 - P_{\rm D}(\Delta t))^n = (1 - P_{\rm D}(\Delta t))^{\frac{T^{\rm C}}{\Delta t}}.$$

Let $m(t)$ be the number of (experimentally) observed cells in a colony at time $t$ and let $m(0) =m_0>0$, then the expectancy for the number of cells after a period $T_{\rm C}$ is given by

29 $$E(m(T^{\rm C})|m(0) = m_0) = 2 (1 - P_{\rm D}(T^{\rm C})) m_0.$$

Let $T_2$ be the experimentally observed time at which the colony has doubled, then

30 $$E(m(T_2)|m(0) = m_0) = \left( 2 (1 - P_{\rm D}(T^{\rm C})) \right) ^{\frac{T_2}{T^{\rm C}}} m_0 = 2 m_0.$$

This implies that

31 $$1 - P_{\rm D}(T^{\rm C}) = 2^{(\frac{1}{T_2} - \frac{1}{T^{\rm C}} ) T^{\rm C}},$$

which, combined with Eq. (28), finally yields the likelihood of cell death over a time-interval $\Delta t$

32 $$P_{\rm D}(\Delta t) = 1 - 2^{(\frac{1}{T_2} - \frac{1}{T^{\rm C}} ) \Delta t}.$$

This relation is used to estimate the probability of death of a cell over a certain time-interval. This likelihood is related to experimental and biological parameters and the length of the time-interval we are interested in. Until now, we did not yet deal with the influence of the environment of the cell on the cell cycle and death.

We postulate that the death and proliferation kinetics of a cell are influenced by the pressure a cell endures. This is in line with the observations that the cells in the core of a tumor experience high pressure, which is part of the reason for the typical necrotic core. Let $p$ denote the pressure due to contact forces that a cell experiences, then we postulate that the probability for a cell to change phase from S to G2 under pressure $p$ is adjusted by

33 $$P_{S\rightarrow {\rm G2}}(p) = \frac{\Delta t}{T^{\rm S}} \cdot F(p), \text { where } F(p) = \frac{c_1}{1+c_2\exp (c_3 p)}.$$

We choose the constants $c_1, c_2$ and $c_3$ such that the ‘average’ cell experiences some pressure and we assert $P_{S\rightarrow {\rm G2}} = 1.1 \frac{\Delta t}{T^{\rm C}}$ if $p = 0$. To this extent, we use $c_1 = 11$, $c_2 = 9$ and $c_3 = 1$. Next to the cell cycle, the pressure also influences the death rate of a cell by

34 $$P_{\rm D}(\Delta t)(p) = 1 - 2^{(\frac{1}{T_2} - \frac{1}{T^{\rm C}} ) \Delta t \cdot G(p)}, \text { where } G(p) = \frac{c_4}{1+\exp (-c_5 p)},$$

for similar consideration as regarding cell cycle, we choose $c_4 = 9$ and $c_5 = 10$.

The tumor cells are modeled as having a higher proliferation rate, that is a faster cell cycle than normal cells, and it is assumed to rather be inclined to adjust its environment than it actually adjusts to its environment. In addition to migration, cell death and cell proliferation, it is possible that tumor cells will also penetrate into the small blood vessels. For simplicity, it is assumed that the stiffness of the extracellular matrix is homogeneous and isotropic. Durotaxis is hence neglected in the present study although we realize that this phenomenon could be of importance. In the model, we assume that tumor cells penetrate into the small blood vessel if the pressure that they apply onto the vessel wall exceeds a certain threshold. We model this in an energetic approach by considering the strain energy density as we did in all the mechanical parts of the model. To this extent, tumor cells penetrate into the small blood vessel with radius $R_{\rm b}$ if

35 $$M({\mathbf{x}}_i) > M_{\rm b}^*, \text { for } i \in {\mathbb{T}}(t).$$

If the above criterion is satisfied then the tumor cell is transported though the blood stream and seeding/spreading of the tumor can later ensue. For each tumor cell that intravasates into the small blood vessel, we assume that the probability of contributing to the spread of the tumor in the body is denoted by $P_S$. Having many tumor cells ending up in the small blood vessel gives an increased probability of seeding out of the tumor, yet the efficiency of tumor spread in the body is very low.

Next to the processes of cell death and proliferation, we take into account the T-cells that enter the domain by transmigration through the small blood vessel walls. The complicated cascade of biochemical cell signaling reactions during this extravasation process is simplified by assuming the probability of a T-cell entering the domain to depend only on the concentration of tumor-cell secreted chemokines. The probability that a T-cell enters the domain is motivated using similar probabilistic principles as earlier, and it is given by

36 $$P_T(\Delta t) = 1 - \left( 1 - \frac{Ac(t,{\mathbf{x}}_{\rm b})^2}{B + c(t,{\mathbf{x}}_{\rm b})^2} \right) ^{\Delta t},$$

where ${\mathbf{x}}_{\rm b}$ denotes a position at a small blood vessel within the domain. These positions have been approximated by a discrete set of points. Proliferation of T-cells is not included and it is assumed that death of a T-cell is described by a stochastic variable as described earlier.

Since tumor cells generally result from mutations due to errors that are made and accumulate during cell division, we incorporate a probability of mutation of a constituent (epithelial) cell describing mutation. This probability per cell division is represented by $P_{\rm M}$. Mutation is modeled at the very last stage of cell proliferation: during the M-phase it is ‘decided’ whether a cell can become a tumor cell. From a biological perspective, mutation occurs during copying DNA, that is during the S-phase, we expect that this adaptation does not significantly contribute to the overall kinetics. The probability of mutation could mimic the lifestyle, gender, or age of the patient. Cell division rate, including the rates characteristic for tumor cells, could mimic the condition and age of the patient.

### The Numerical Method and Parameter Values

In this subsection, we describe the numerical method and parameter values.

#### Time-Integration Method

For the solution of the stochastic differential equations that describe cell migration, we use the fourth order Runge–Kutta method for the deterministic part. For the stochastic part, a classical Maruyama method is used. The time-step is limited such that a cell maximally displaces over a quarter of its diameter. This implies that the numerical time-step often fluctuates over the simulation time. The cell growth during the cell cycle is dealt with analogously. The concentration of cytokines that is obtained through integration over time is determined using the Trapezoidal Rule for numerical quadrature. Note that the history-path of the tumor cell locations have to be stored during the entire simulation. We also have to take into account the contributions from the tumor cells that already died. Since it can be proved that these contributions decrease monotonically over time down to zero and that hence after a certain amount of time these contributions become negligible, the terms are disregarded if they get lower than a certain tolerance. From our simulations, we saw that the effects from already dead tumor cells become negligible as soon as $t -\tau _{\rm D} \ge \tilde{t}$. This time $\tilde{t}$ depends on the problem, parameter values* etc.* In our simulations, we use $\tilde{t} = 80$ where contributions to the chemokines concentration from already dead tumor cells became less than a tenth of a percent, which is considered negligible. For even more gaining of computational efficiency, we consider the function $C(s)$ for large times with various distances between the position ${\mathbf{x}}$ and the location of a tumor cell ${\mathbf{x}}_k$. For long times, the concentration of chemokines released by tumor cell $k$ can be approximated by integrating over

37 $$C(s) = \frac{\gamma }{4 \pi D (t-s)},$$

which gives

38 $$\begin{aligned} \int _{\tau _B}^{t-t^*} C(s) ds = {\left\{ \begin{array}{ll} \displaystyle {\frac{\gamma }{4 \pi D} \log \left(\frac{t-\tau _B}{t^*}\right)}, &{} \text { in } {\mathbb{R}}^2,\\ \displaystyle {\frac{\gamma }{2 \pi D} \left( \frac{1}{\sqrt{t^*}} - \frac{1}{\sqrt{t-\tau _B}} \right) }, &{} \text { in } {\mathbb{R}}^3. \end{array}\right. } \end{aligned}$$

Here we assumed for simplicity that $\gamma$ does not depend on time. A temporal dependence could be dealt with analogously in deriving just upper bounds. These aforementioned rules are used to approximate the concentrations at long times on the positions of the points on the small blood vessel. To gain a further reduction in integration time, we use time-steps in the integration of the concentrations that are larger than the time-steps in the migration, death, and proliferation of cells. As the overall time-step decreases down to zero, this time-step also decreases down to zero. Note that these concentrations are only needed at the positions of the T-cells and on the points on small blood vessels where T-cells possibly originate from. At no other points are these concentrations computed. This is opposite to discretisation methods like the finite-element method where global concentration fields have to be computed.

#### Parameter Values

To perform the simulations, we use the values that are listed in Table 1. Most of the parameter values were not known and since our paper only aims at the formulation of a model, we have chosen the parameter values such that they mimic reality as well as possible. We note that for the radius of cells in the S-phase, we used the arithmetic mean of the initial cell radius right after cell division and the cell radius right before division. The average residence times of cell in the subsequent phases are listed for epithelial cells and tumor cells. On the basis of these times, the probability of cell death is estimated using the principles from Section 2.3. We note that these times should be considered as dimensionless and hence do not reflect quantitative reality. They merely reflect the proportion of the cell cycle times of the tumor cells in relation with the cell cycle times of the epithelial cells. The times in Table 2 for the proliferation rates of the tumor cells have been exaggerated so that a fast tumor development is predicted to illustrate the potential of the model.

**Table 1:** Default parameter values.

Parameter	Value	Unit
$E_{\rm s}$	5.0	kPa
$E_{\rm c}$	0.5	kPa
$\tilde{F}$	1	nN
$M_{\rm b}^*$	0.1	kPa
$R_0$	3	*μ*m
$R_{\rm max}$	$2^{1/3} R_0$	*μ*m
$R_{\rm T}{\text {-}}{\rm cell}$	4.5	*μ*m
$R_{\rm b}$	4	*μ*m
$L$	30	*μ*m
$D$	100	$\mu {\rm m}^2 {\rm s}^{-1}$
$\gamma$	2	nmol/(m^3^ s)
$f$	0.2	–
$\beta _i$	10	s^−1^
$\mu$	$10^4 \alpha _i$	–
$P_{\rm M}$	0.25	–
$A$	0.05	–
$B$	200	–

**Table 2:** Average residence times (s).

Parameter	Epithelial cell	Tumor cell
$T_{\rm G1}$	300	10
$T^{\rm S}$	400	0
$T_{\rm G2}$	300	10
$T_{\rm M}$	1	1
$T^{\rm C}$	1,001	21
$T_2$	$10^7$	4,000

## Results

We show how the model works in the two-and three-dimensional case. We vary the parameter values in a two-dimensional setting since the computations need more time in the three-dimensional case.

### Two-Dimensional Simulations

**Figure 2:**
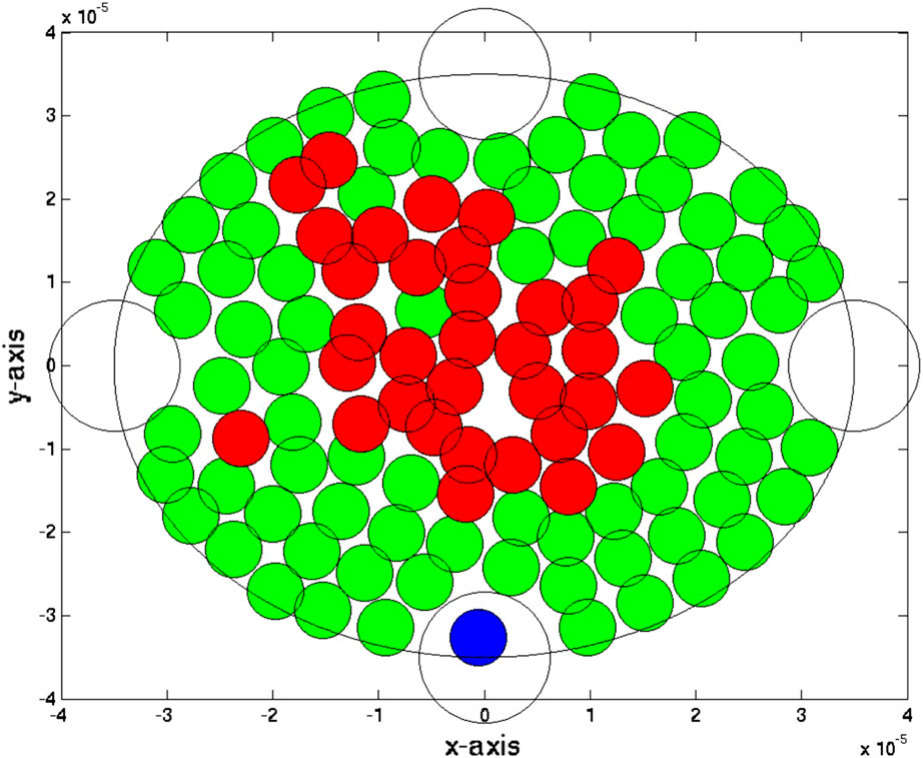
A snapshot of the cell composition and arrangement of the cells in the tissue. The green cells denote the epithelial cells, the red ones are the tumor cells and finally the blue cells are the T-cells (not present here). Further, the large white circles represent the small blood vessels or the points at which T-cells possibly originate.

**Figure 3:**
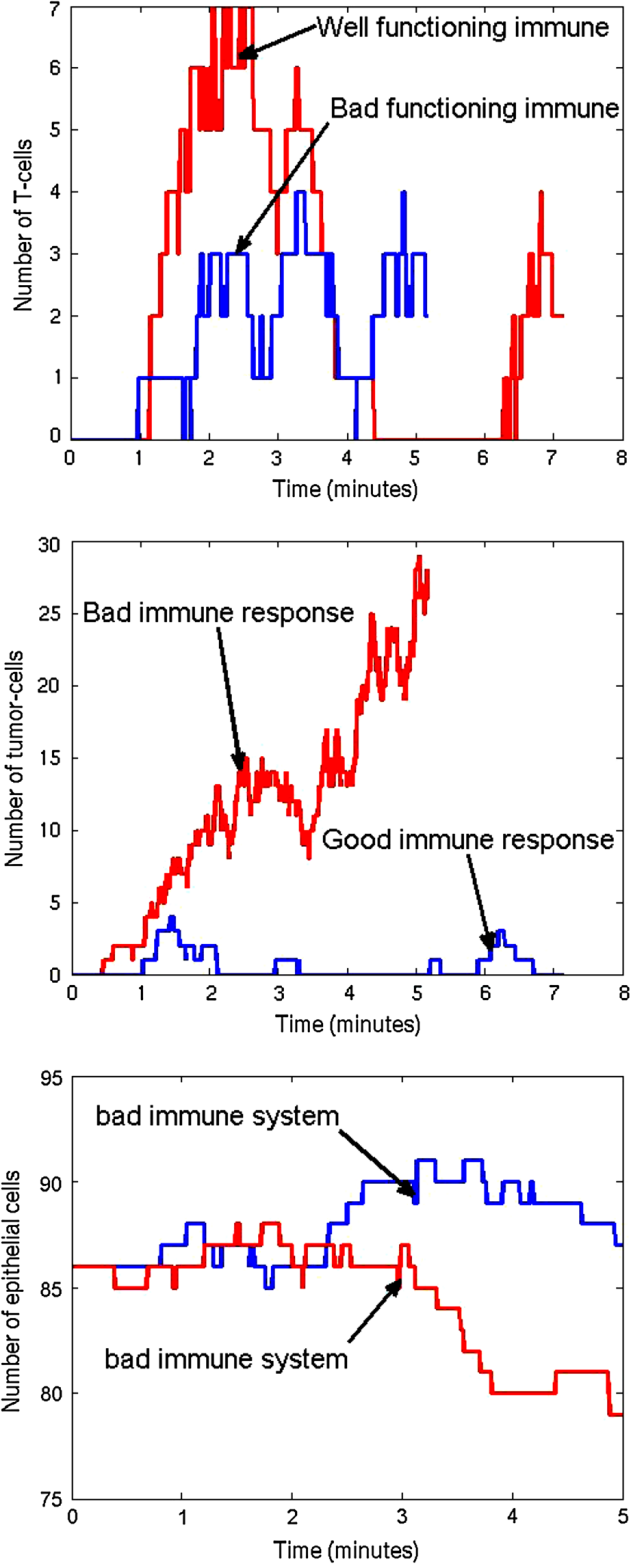
The number of T-cell counts (left), tumor cells (middle) and epithelial cells (right) in the tissue vs. time for a ‘good’ and ‘bad’ (with adjusted immune system parameters).

**Figure 4:**
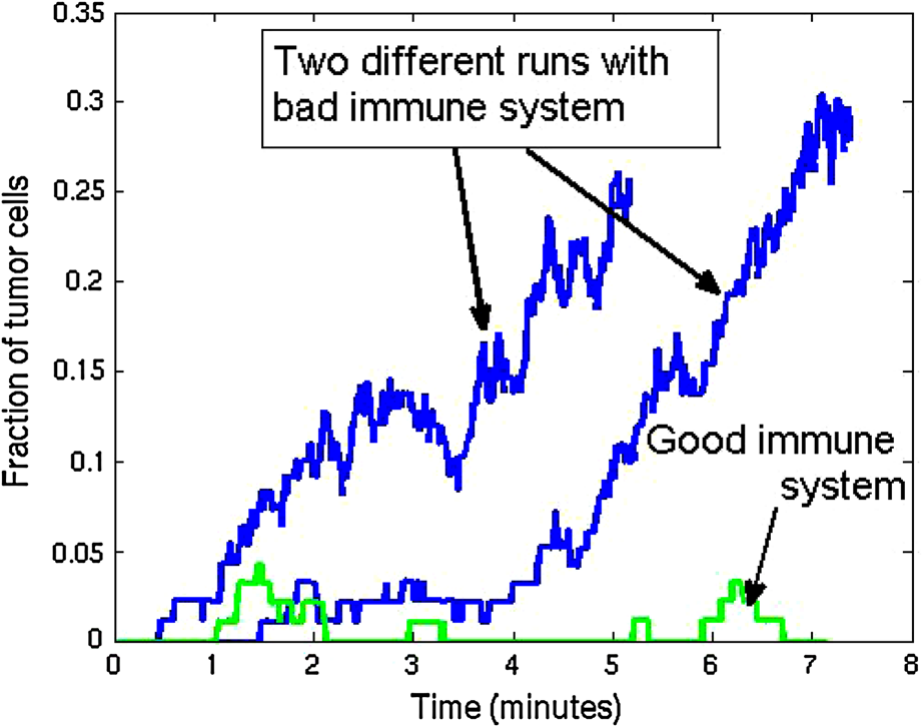
The number of tumor cells in the tissue vs. time for a ‘good’ and ‘bad’ (with adjusted immune system parameters). For the ‘bad’ immune system case, we show two different runs.

We start illustrating the method in two spatial dimensions. In Fig. 2 we show a snapshot of the considered tissue where we consider a domain with radius of 40 micrometer that is initially filled with endothelial cells. On four points of the domain, we assume that T-cells can invade the region where they neutralize the tumor cells. The epithelial cells are depicted as green cicles, whereas the tumor cells and T-cells, respectively, are represented by red and blue circles. We use the data from Tables 1 and 2 as input data, and we adjust the data for the immune system, where we increase the T-cell mobility, and appearance rate on the small blood vessels. To this extent, we model a ‘good’ immune system response by taking $A = 0.5$, $B = 100$ and $\mu = 5 \times 10^4 \alpha _i$. The results for the T-cell counts are given in Fig. 3. It is clear that the T-cell counts are significantly higher for the ‘good’ case. Further, it is also clear to see that the number of tumor cells significantly reduces with a ‘good’ immune system response as to be expected. The ‘good’ immune system is able to battle the tumor in its early stages and the tumor cells disappear completely. It is further clear to see that the number of epithelial cells stays more or less constant with a ‘good’ immune system response, whereas if the immune system is poor then the number of epithelial cells decreases due to enhanced cell death as a result of the pressure that is experienced by the epithelial cells. To show the variation from the uncertainty in the model, we show the tumor cell fraction for two runs with identical parameters for the ‘bad’ immune system response, and one run with a ‘good’ immune system in Fig. 4. Although the variations between the two ‘bad’ immune system response are considerable, we see that the fraction ranges up to about 30 percent in both runs. Hence qualitatively the runs mimic similar behavior. The results for the ‘good’ immune system mimic a totally different behavior for the tumor cell fraction. The fraction stays around zero at all times. From these calculations, it is shown that a good immune system response could prevent cancers to initiate.

### Three-Dimensional Simulations

**Figure 5:**
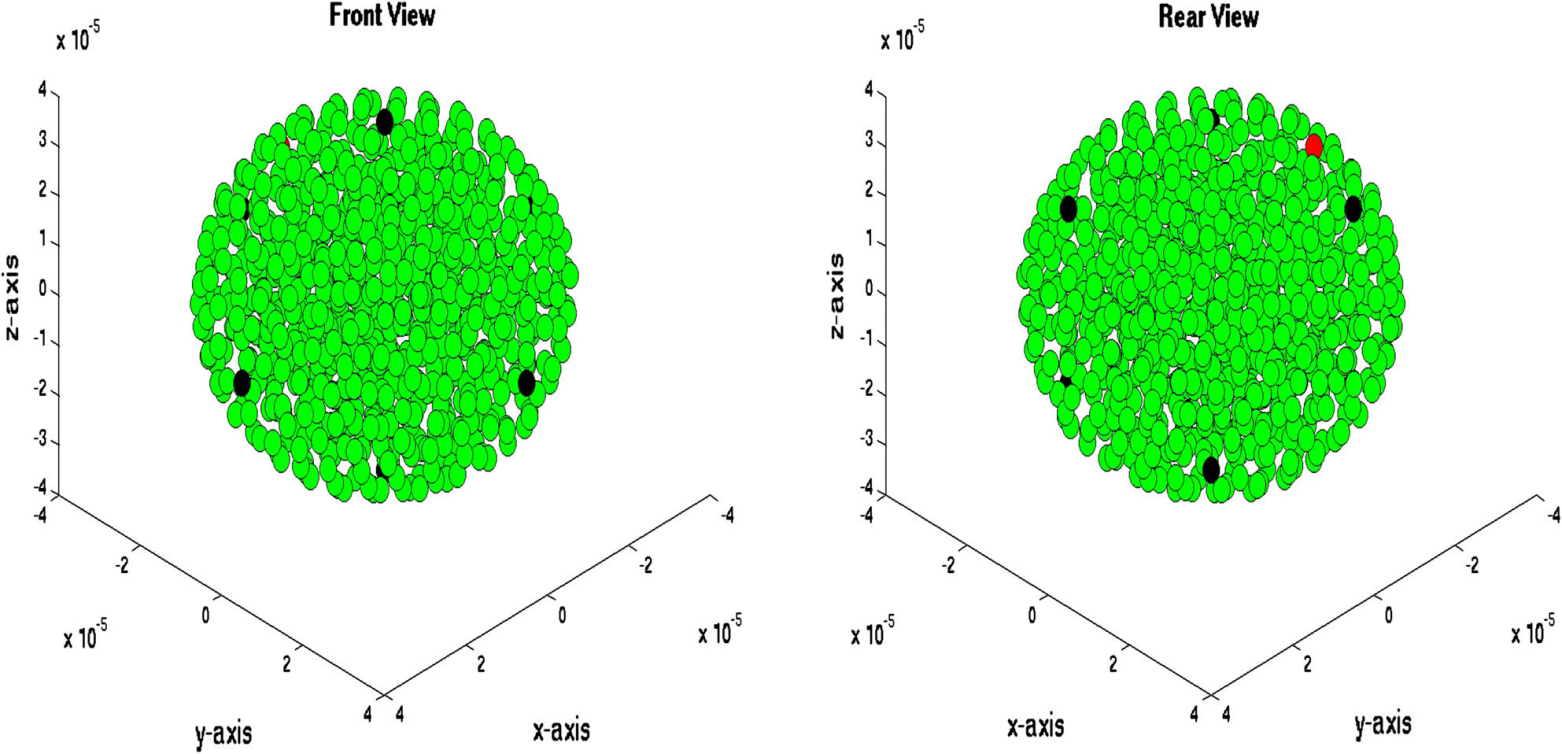
The stage just after the initial state for the simulation of tumor development. The green cells denote the epithelial cells, the red ones are the tumor cells and finally the blue cells are the T-cells (not present here). Further, the large black dots represent the points at which T-cells possibly originate. Left: front view, Right: rear view.

**Figure 6:**
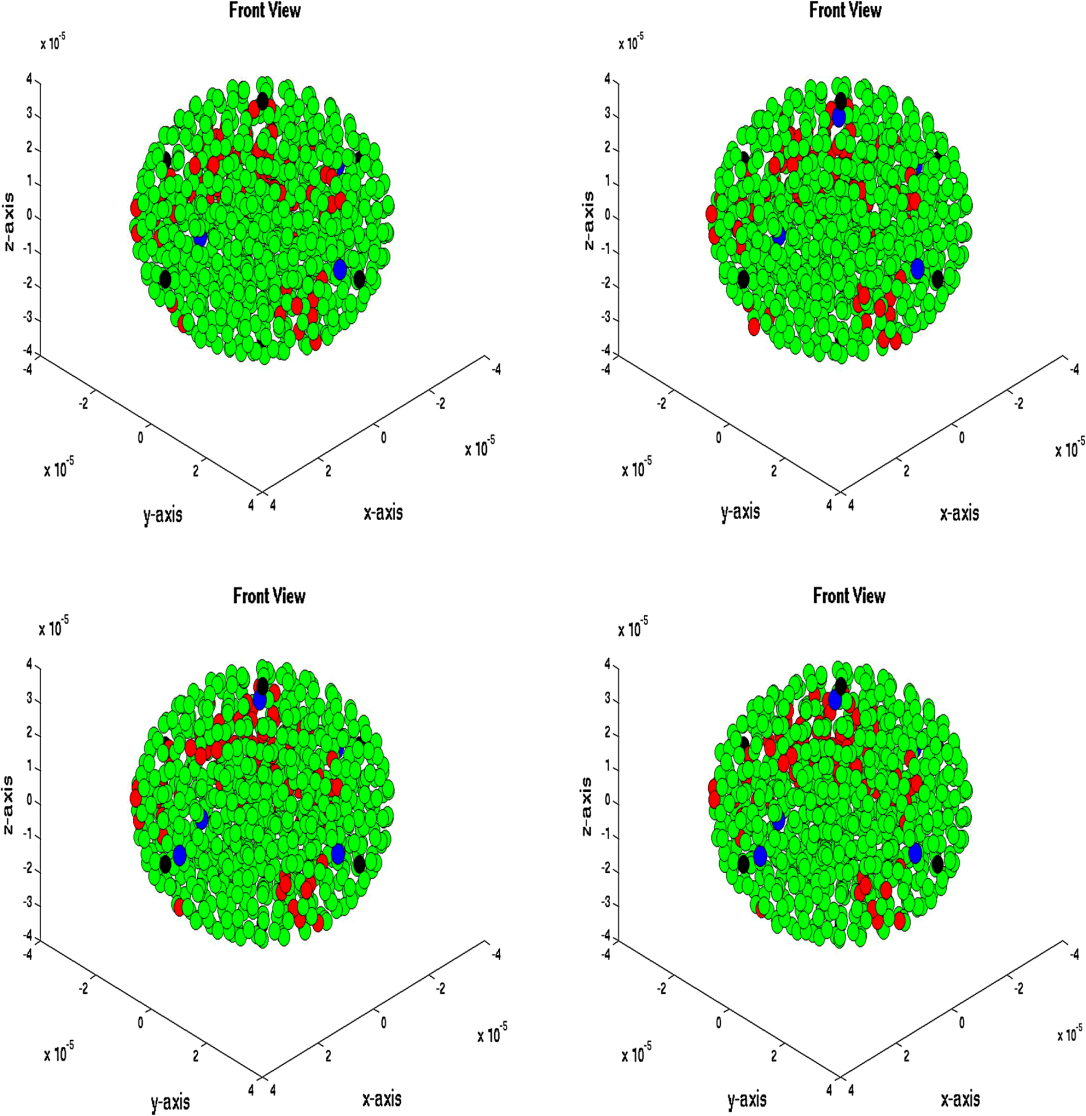
Front view of tumor development at four consecutive times. The green cells denote the epithelial cells, the red ones are the tumor cells and finally the blue cells are the T-cells (not present here). Further, the large black dots represent the points at which T-cells possibly originate. Snapshots are at times 128,130,132 and 134 s.

**Figure 7:**
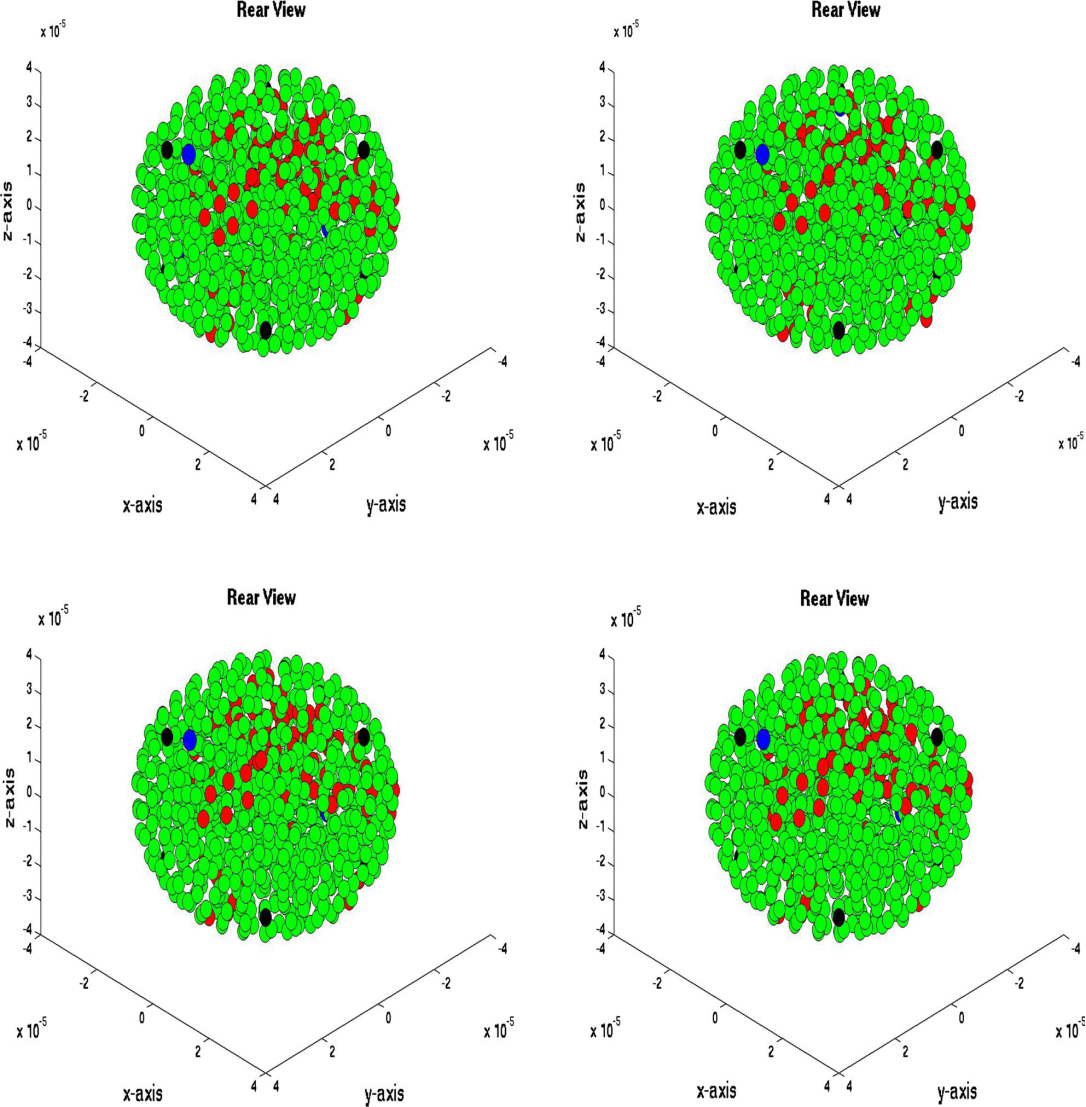
Rear view of tumor development at four consecutive times. The green cells denote the epithelial cells, the red ones are the tumor cells and finally the blue cells are the T-cells (not present here). Further, the large black dots represent the points at which T-cells possibly originate. Snapshots are at times 128,130,132 and 134 s.

**Figure 8:**
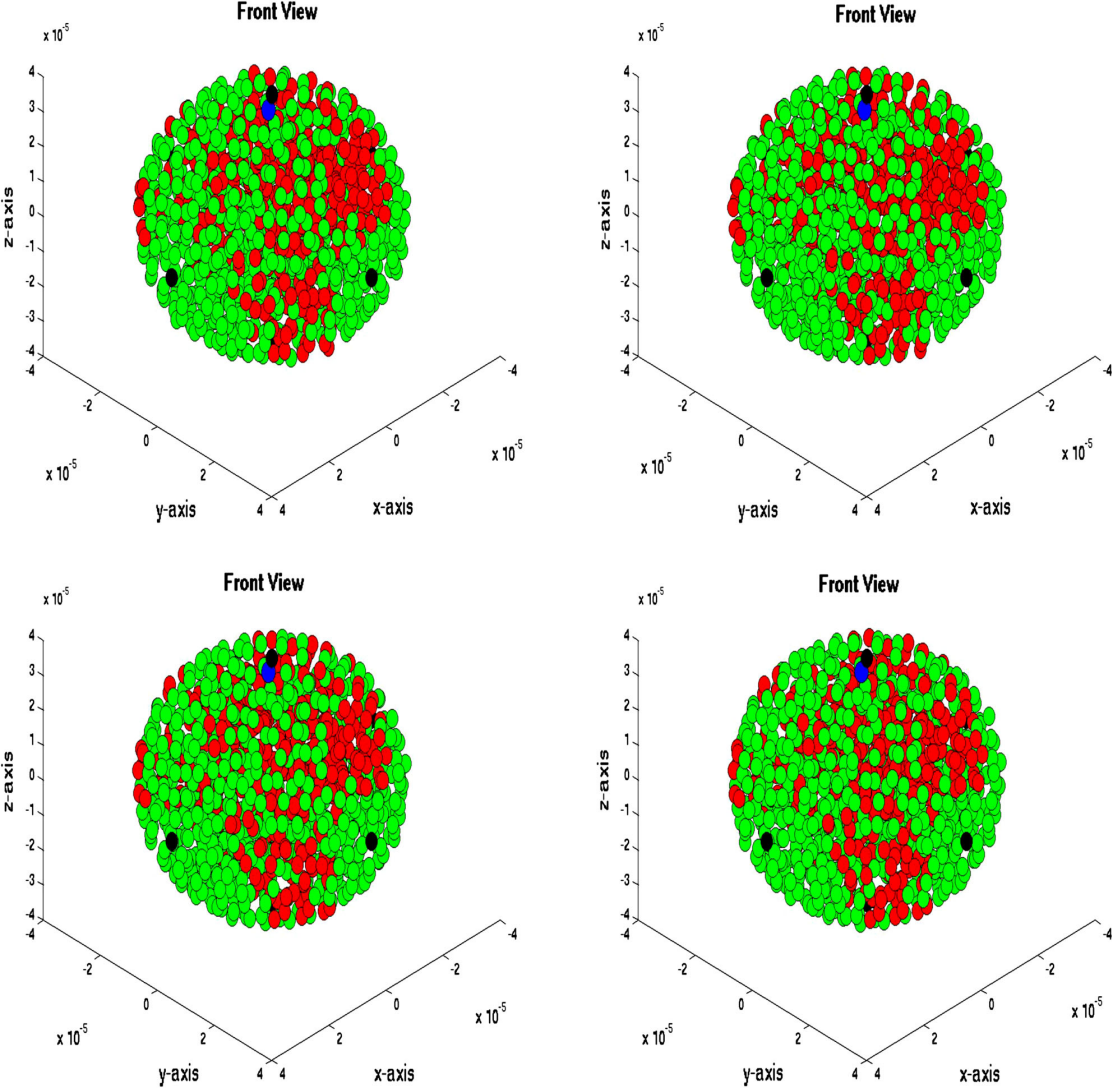
Front view of tumor development at four consecutive times at a later stage. The green cells denote the epithelial cells, the red ones are the tumor cells and finally the blue cells are the T-cells (not present here). Further, the large black dots represent the points at which T-cells possibly originate. Snapshots are at times 494, 496, 498 and 500 s.

**Figure 9:**
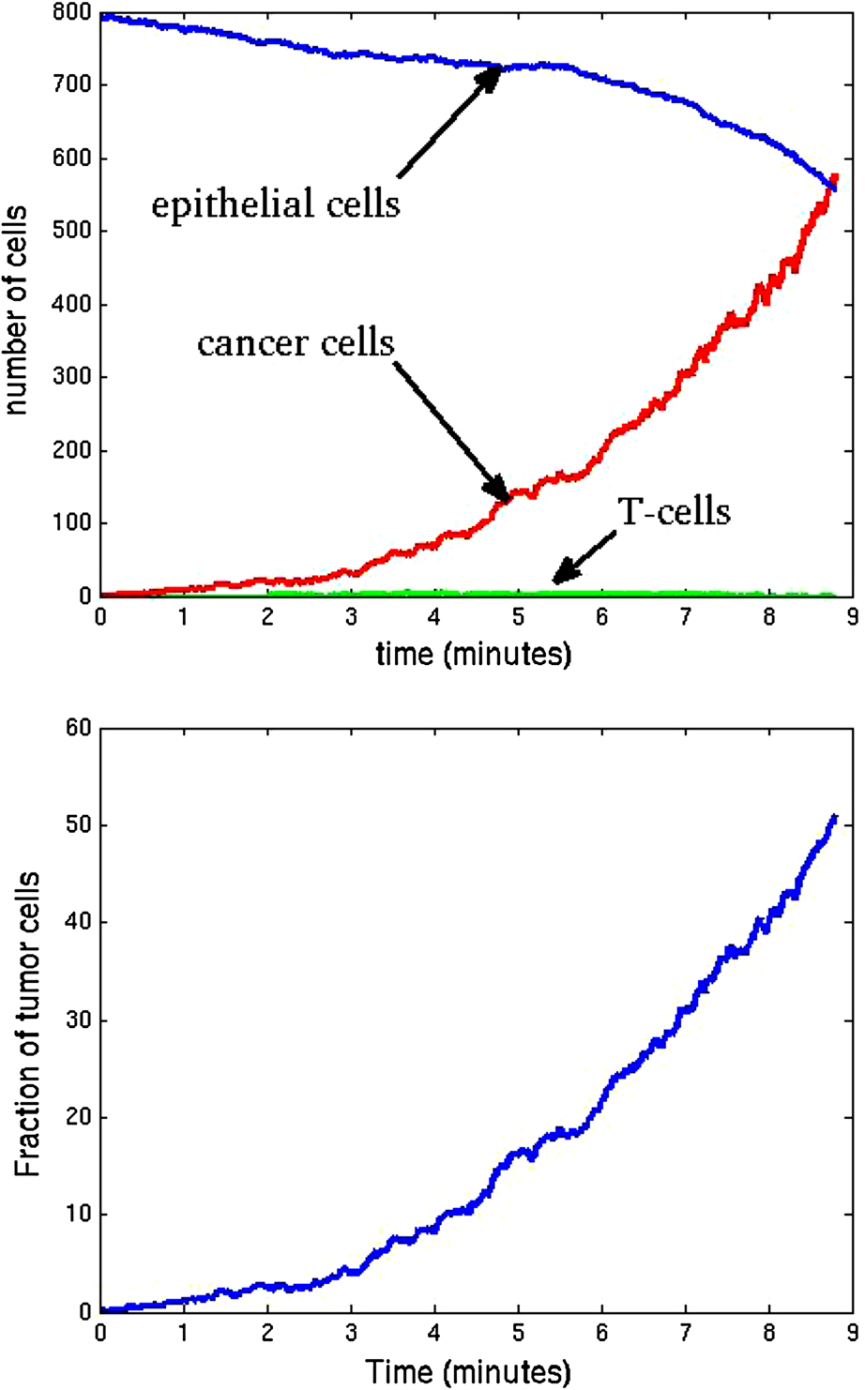
Left: Plot of the number of cells vs. time in days. Represented are the number of tumor-cells, epithelial cells, and T-cells vs. time. Right: the fraction of tumor cells as a function of time.

**Figure 10:**
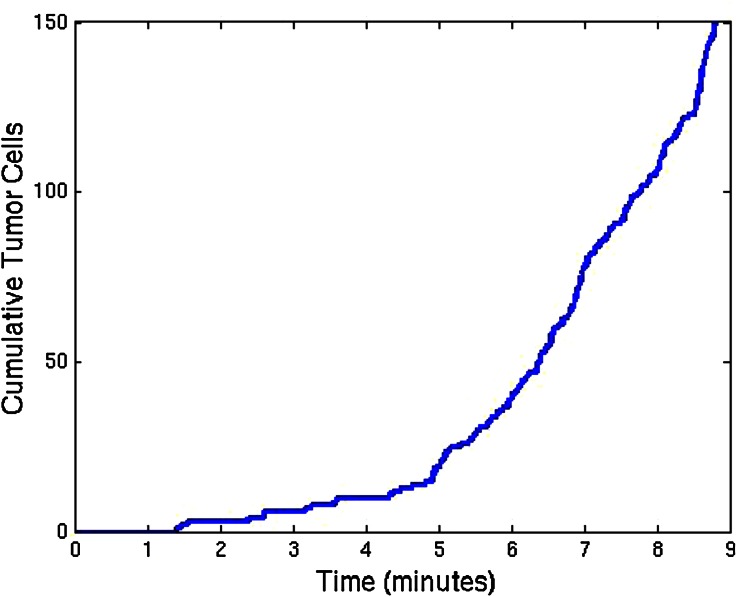
Plot of the accumulated number of tumor-cells that transmigrate into a small blood vessel vs. time in days. Transmigration takes place if the pressure of the tumor-cell onto the vessel wall exceeds a certain threshold value.

We consider a spherical domain with radius of 40 micrometer that is initially filled with endothelial cells. The epithelial cells are allowed to migrate, proliferate and allowed to die. Further, they may mutate to tumor cells. At six points of the domain edge, we assume that T-cells can invade the domain. The epithelial cells are depicted as green spheres, the tumor cell are red and finally the T-cells are blue spheres. Further, the small blood vessel points where T-cells originate from are depicted by large black dots. See Fig. 5 for the initial state. At later stages, it can be seen that the T-cells appear in the domain and start chasing the tumor cells. To this extent, we plot four consecutive snapshots of the front and rear view in Figs. 6 and 7. In Figs. 6 and 7, it can be seen that the tumor develops in a continuous way. Tumor cells are seen to be inside the cell colony, where the T-cells invade the region from the boundary. The T-cells have to make their way by chemotaxis while enduring the contact forces that they experience from the native epithelial cells. In Fig. 8, we see the cell pattern at a late stage where the tumor cells are dominant in the tissue. The number of red tumor-cells has increased tremendously. This stage could account for a decrease of performance of the body under consideration. One could insert a measure for disfunction of an organ to quantify the damage that the tumor is causing to the patient. Further, in Fig. 9, we plot the number of T-cells, tumor cells and epithelial cells vs. time, from which the proportion of the tumor cells can be determined, see also in Fig. 9. It can be seen that the calculation was stopped near the point where the fraction of the tumor cells is equal to 0.5 (that is 50 %). The number of tumor-cells increases almost exponentially and gradually take over the tissue by making the epithelial cell counts decrease as a result of the pressure that is exerted onto the latter. The number of T-cells does not increase sufficiently in this run to fight the tumor cells. Next to plotting the number of cell counts for the various phenotypes over time, we present the cumulative number of tumor-cells that transmigrate into the small blood vessel in Fig. 10. It can be seen that this number increases exponentially. This number of cells is related to the likelihood of tumor expansion into other anatomic parts of the body. The exponential nature of this quantity over time illustrates the danger of tumor expansion, which is partly kept in check by the immune system and various obstacles that the cells encounter.

## Discussion

A mathematical model for the initiation of tumors has been formulated in terms of hybrid modeling where all cell types (epithelial cells, tumor-cells and T-cells) have been treated individually, whereas the concentration of tumor-released chemokines through the tissue matrix is modeled by solving the diffusion equation on a continuum. The stiffness of the extracellular matrix is taken into account in the traction forces that the cells ‘colorred exert. We, however, assume that the stiffness does not change in time, despite the fact that cancer cells do change the stiffness of their environment. This feature will be dealt with in future studies. Cells are allowed to mutate, proliferate, die and to migrate by chemotaxis, haptotaxis, and random walk. The cell shape is spherical and circular in the three-and two dimensional simulations, respectively. The chemokines are monitored by the use of Green’s Fundamental solutions, which allows the determination of the concentrations only at these points where they are needed without the need of solving the entire diffusion field using discretisation techniques like the finite-element method. The method does, however, need elaborate time-integration procedures for the evaluation of the Green’s Functions and to make the computations tractable we investigate the increase of numerical efficiency under retaining reasonable (qualitative) accuracy levels. The Fundamental solutions are valid over an infinite domain, which simplifies the approach. On the other hand, finite-sized domains require the formulation of boundary conditions such as periodic conditions or no-flow conditions where these conditions are required to describe physical cases. Since the possible diffusion field is much larger than the physical domain in many cases, the mathematical need for describing boundary conditions is therefore not always desirable. We note that linear, isotropic diffusion is a simplification since diffusivities often depends on the composition of the extracellular matrix. We also remark that diffusion in many tissues could be non-isotropic. Non-isotropic diffusion would need either a finite-element approach or could be tackled with the use of diagonalization arguments combined with the Fundamental solutions. The latter approach could be an interesting topic of further research. These simplifications enable the use of the Green’s Fundamental solutions. Furthermore, as mentioned earlier the changes that are inflicted on the extracellular matrix by the tumor cells are disregarded in the present study. We also realize that the mechanical issues in the model are simplified by the use of a phenomenological relation for the displacements and strain energy density. The present approach enables to get rapid numerical results and a good qualitative reproduction of the mechanical signals and its consequences. Boundary effects, as well as non-isotropic effects need a different finite-element based approach, which would pose a major revision of the mechanical part of the model.

The number of cell-types including tumor cells is limited to three phenotypes, which is a simplification of reality. We also bear in mind that in this sense the model exactly captures the most important features like spread and proliferation of tumor-cells at the expense of the constituent epithelial cells, and thereby the model performs very well. The mechanism of T-cells transmigration through the small blood vessel walls, is simplified in the current model. Although the entire chain of bio-chemical reactions could be taken into account, this model extension will not change the nature of the modeling results and implications, but would rather make the model less tractable in the sense that the complexity increases, which faces us with the need of more bio-physical parameters to determine from possible experiments.

In this sense, having a minimalistic model that still captures the most relevant aspects of the initiation of tumors: through cell proliferation and migration and even captures the most important qualitative aspects of the performance of the immune system response to tumor development, is a very valuable tool. The model, although still in its developmental phase, can be used to monitor the use of drugs and other treatments to battle cancer development. Even life-style of a patient could be varied in the simulations for the evaluation of the likelihood of cancer development. One could think of certain underlying diseases that could increase the probability of mutations, as could prescription drugs or environmental conditions. Extension to larger cell numbers is mathematically straightforward, but requires the use parallel algorithms, such as executed in a Graphical Processing Unit (GPU) environment. This will be done in future work and this will also be key to real-world geometries and tissue applications.

**Figure 11:**
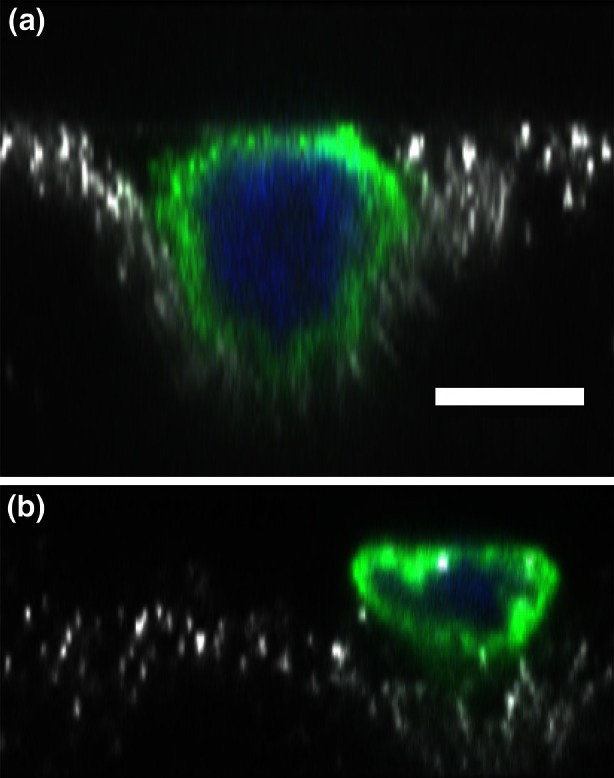
Side view using confocal imaging of cells interacting with a impenetrable, polyacrylamide gel substrate with elastic modulus of 2300 Pa. The white are particles marking the gel surface location, green is microtubules, and blue is the cell nucleus. A A low metastatic potential breast cancer cell (MDA-MB-468) applies pressure to the substrate and indents it, likely in attempted penetration. B A benign breast cell (MCF-10A) will adhere to the substrate, flatten out slightly, but will not significantly deform the substrate. Scale bar is 10* μ*m.

In addition to tumor initiation, the model is capable of monitoring the pressure applied by the tumor-cells on the small blood vessel walls. If the pressure exceeds a certain threshold value, then the cell is allowed to penetrate into the vessel wall, which is assigned a quantitative likelihood for the successful spreading of an initiated tumor at other organs in the body. Figure 11 shows that while cancer cells will attempt to invade a soft substrate, benign cells will simply attach to it.

Finally, we realize that the stochastic nature of the model requires the execution of multiple simulations for each parameter set. In future studies, these natural model variations will be studied more extensively once a solid parallel computational framework has been accomplished.
